# Are membranes non-apoptotic compartments for apoptotic caspases?

**DOI:** 10.18632/oncotarget.25796

**Published:** 2018-08-03

**Authors:** Andreas Bergmann

**Affiliations:** Department of Molecular, Cell and Cancer Biology, University of Massachusetts Medical School, Worcester, MA, USA

**Keywords:** apoptosis-induced proliferation, undead cells, caspase, Myo1D, plasma membrane

**Figure 1:**
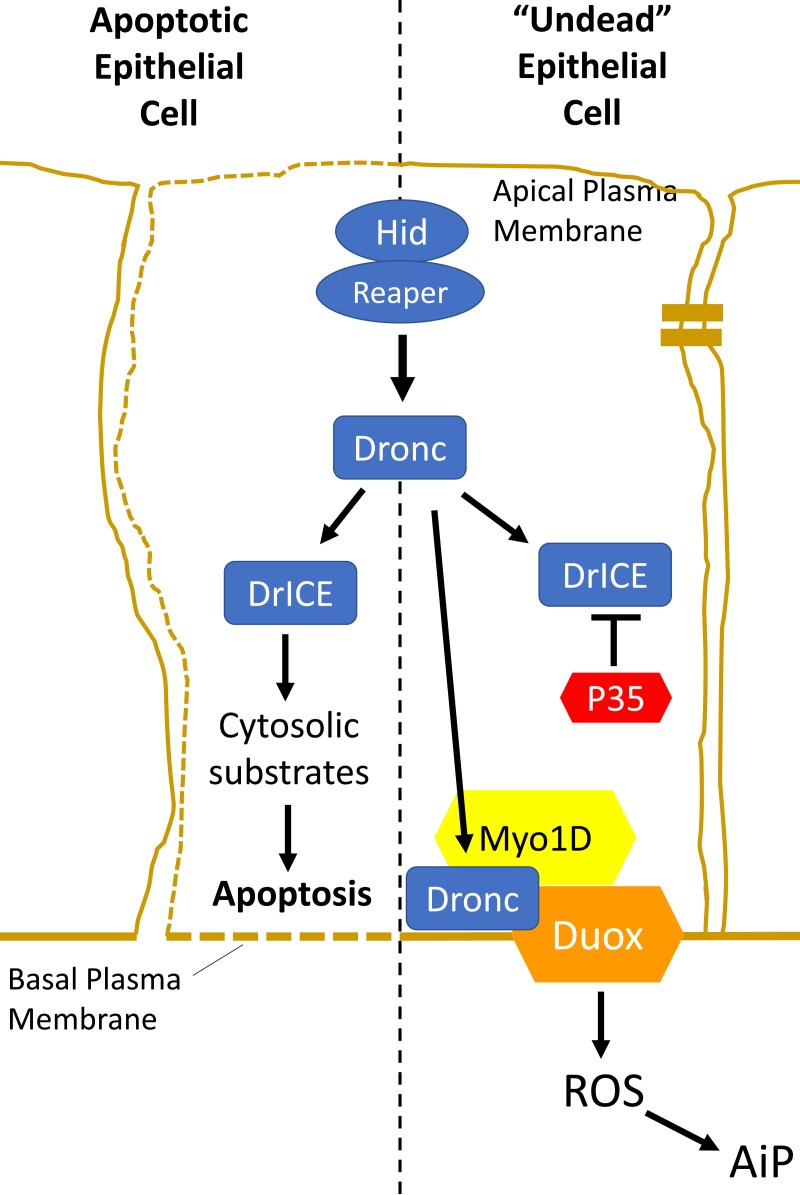
Activation of the apoptotic pathway in apoptotic and “undead” epithelial cells has different outcomes In both cases, the apoptotic pathway is induced upstream by expression of the pro-apoptotic genes *hid* or *reaper*. That triggers the activation of the caspase-9-like initiator caspase Dronc. The outcome of Dronc activity is different in both cases. Left. In an apoptotic epithelial cell, cytosolic Dronc promotes the activation of the caspase-3 ortholog DrICE which cleaves a large number of cytosolic proteins, triggering apoptosis. Right. In an undead epithelial cell, the execution of apoptosis is blocked by expression of P35 (red). Dronc is translocated to the basal side of the plasma membrane by the unconventional myosin Myo1D (yellow) where it directly or indirectly activates Duox (orange) for the generation of extracellular ROS required of apoptosis-induced proliferation (AiP).

Critical mediators of apoptotic cell death are caspases, a highly specialized class of Cys-proteases that cleave substrates after Asp residues. Under normal conditions, caspases are cytosolic proteins. After their activation, they cleave a large number of cytosolic proteins and execute apoptosis (Figure 1, left). However, in addition to their well-studied role in apoptosis, caspases also have many non-apoptotic functions [1, 2]. It is not very well understood how cells escape the potential harmful action of caspases when they perform non-apoptotic functions. In our recent work, we now show that epithelial cells may prevent apoptosis by sequestration of caspases at the plasma membrane, specifically the basal side of the plasma membrane, for non-apoptotic functions [3].

We are studying the role of caspases for the generation of mitotic signals to neighboring surviving cells independently of their apoptotic function. This type of compensatory proliferation has been termed Apoptosis-induced Proliferation (AiP) [4]. Work in several model organisms (*Drosophila*, Hydra, Planaria, Xenopus, Zebrafish and Mouse) has revealed that AiP is involved in tissue homeostasis, regeneration and cancer (reviewed by [5]. We are using the genetic model organism *Drosophila melanogaster* to examine AiP.

To identify the genes and mechanisms of AiP, we are taking advantage of a genetic trick. It is known that the initiator caspase Dronc (Caspase-9 ortholog in *Drosophila*) is required for AiP. Therefore, we are inducing apoptosis upstream of Dronc by expression of the pro-apoptotic genes *hid* or *reaper*, but block apoptosis downstream of Dronc by co-expression of the effector caspase inhibitor *p35* which specifically inhibits the caspase-3 ortholog DrICE (Figure 1, right). Under these conditions, the apoptotic pathway is activated, but it cannot execute apoptosis, rendering cells in a so-called “undead” condition. Dronc is not inhibited by p35 and can continuously signal for AiP in the absence of apoptosis resulting in tissue overgrowth if undead cells are produced in epithelial cells of imaginal discs.

We are performing genetic modifier screens of the undead overgrowth phenotype to identify genes and mechanisms of undead AiP [6]. One of these genes is the plasma membrane-bound NADPH oxidase Duox which produces extracellular reactive oxygen species (eROS) (Figure 1, right). Removing eROS by *Duox* RNAi or expression of extracellular catalases blocks AiP demonstrating the important role of Duox and eROS for AiP [7]. eROS attract and activate hemocytes, *Drosophila* immune cells of the macrophage lineage, which are also required for AiP [7].

The question that we were addressing in our recent work is how Dronc activates the NADPH oxidase Duox. In our genetic screens, we have identified another gene, the unconventional myosin, *Myo1D*, as essential for undead AiP. Previously, Myo1D has been implicated in left/right (L/R) development in *Drosophila*. Although *Drosophila* is a bilateral organism, the development of certain visceral organs such as the coiling of the gut and a very peculiar morphogenetic movement of the male genitalia (male genitalia rotation) during pupal development occurs in a certain L/R asymmetric way. Interestingly, the cell death pathway has also been implicated in male genitalia rotation [8], so the identification of Myo1D in AiP did not come as a total surprise. However, how Myo1D and the apoptotic pathway are linked was completely unknown.

Mechanistically, Myo1D physically interacts with the initiator caspase Dronc, but it is not a cleavage substrate of Dronc. Instead, Myo1D is required for localization of Dronc to the plasma membrane of undead epithelial cells which brings Dronc in close proximity to the NADPH oxidase Duox [3]. Interestingly, the membrane localization of Dronc by Myo1D occurs only at the basal side of the plasma membrane (Figure 1, right). This makes sense because Duox is also only localized at the basal side of undead epithelial cells and hemocytes are recruited to imaginal discs at the basal site [3].

Importantly, we also demonstrated that the activity of Dronc at the basal plasma membrane is non-apoptotic in nature. We propose that the basal site of the plasma membrane constitutes a non-apoptotic compartment for caspases to fulfil non-apoptotic functions. It is possible that at the plasma membrane, Dronc in particular and caspases in general may be sequestered away from apoptotic substrates which allows caspases to perform non-apoptotic functions without killing the cell. It is also possible that the apoptosome partner of Dronc, termed Dark (*Drosophila* Apaf-1 related killer), is not very abundant at the plasma membrane keeping the overall caspase activity inside the cell below the threshold needed for apoptosis to occur. Future work will reveal the mechanisms by which active caspases localized at membranes and at other non-apoptotic compartments leave the cells intact.
